# Sensing-Aided Communication Method for Distributed Radar Communication System

**DOI:** 10.3390/s25103028

**Published:** 2025-05-11

**Authors:** Xinren Wang, Sisi Xia, Zhongzheng Ding, Qin Wang, Wenchao Xia, Haitao Zhao

**Affiliations:** 1 Portland Institute, Nanjing University of Posts and Telecommunications, Nanjing 210023, China; p22000506@njupt.edu.cn; 2 Institute of Internet of Things, Nanjing University of Posts and Telecommunications, Nanjing 210003, China; b22130808@njupt.edu.cn (S.X.); zhongzheng0721@163.com (Z.D.); wangqin@njupt.edu.cn (Q.W.); xiawenchao@njupt.edu.cn (W.X.); 3 School of Communications and Information Engineering, Nanjing University of Posts and Telecommunications, Nanjing 210046, China; 4 School of Internet of Things, Nanjing University of Posts and Telecommunications, Nanjing 210003, China

**Keywords:** distributed radar communication system, integrated sensing and communication (ISAC), dynamic power allocation, multi-user interference, user mobility

## Abstract

Integrated Sensing and Communication (ISAC) has emerged as a key direction in 6G technology and is currently a major focus of research. This paper proposes a dynamic power allocation scheme that, under the constraints of transmission power and detection probability, formulates power allocation as an optimization problem aimed at maximizing the system sum rate. The approach accounts for varying target motion states, inter-user interference, and channel conditions. By jointly considering target motion sensing, inter-user interference, and channel conditions, our approach outperforms traditional methods that optimize communication and sensing separately. Numerical simulation results show that this scheme can achieve excellent performance in complex real-world environments. This work contributes to the development of efficient ISAC solutions and provides insights for future research into 6G technologies.

## 1. Introduction

The rapid evolution of wireless networks and sensing technologies has led to the emergence of Integrated Sensing and Communication (ISAC) as a key enabler of 6G communications [1,2,3]. ISAC systems aim to unify wireless communication and radar sensing, allowing both functions to coexist in a shared spectrum, which improves spectral efficiency, energy utilization, and real-time adaptability [4,5]. This paradigm is essential for applications such as autonomous vehicles, smart cities, military surveillance, and industrial automation.

In distributed radar communication systems, resource allocation poses a fundamental challenge. Traditional radar and communication networks operate independently, leading to inefficient spectrum usage and excessive power consumption [6]. The demand for optimal spectrum sharing has led to research into power allocation strategies that maximize communication rates while ensuring detection precision [7]. Earlier studies explored waveform optimization and beamforming techniques to balance communication and sensing [8,9,10]; however, these approaches have shown limited effectiveness in scenarios involving large and moving users in complex urban environments, i.e., user mobility and inter-user interference.

Inter-user interference is another major issue, especially in distributed networks where multiple users share the same spectrum [11]. Conventional interference mitigation methods, such as game theory-based power control and water-filling power allocation [12], provide suboptimal results in highly dynamic environments. Although machine learning-based approaches have also been explored for interference prediction, their high computational cost limits real-time applications.

Many scholars have conducted extensive research on Integrated Sensing and Communication (ISAC) technologies. For example, Dou et al. introduced a sensing-efficient NOMA-aided ISAC method, optimizing joint sensing scheduling and beamforming to significantly enhance resource utilization efficiency [13]. Huang et al. developed an edge intelligence-oriented ISAC framework, proposing multi-cell cooperative strategies to bolster network robustness and performance in complex scenarios [14]. Additionally, Zhang et al. explored terahertz-enabled UAV-based ISAC systems, emphasizing advanced transceiver designs aimed at improving the integration and performance of communication and sensing functionalities in future 6G networks [15]. Xia et al. studied multi-armed bandit-based client scheduling methods for federated learning, enhancing client selection efficiency and overall system performance in wireless communication contexts [16]. Bazzi et al. proposed a low dynamic range design for RIS-aided bistatic ISAC systems, leveraging array signal processing and interference optimization techniques to enhance SNR and system reliability under 6G communication constraints [17]. Furthermore, Xia et al. proposed federated-learning-based scheduling schemes specifically tailored for low-latency wireless communication applications, effectively reducing communication latency and improving network efficiency [18].

The industry has conducted an in-depth exploration to design the performance index of integrated 6G communication and sensing systems. When implementing the dual functions of communication and perception in the system, it is often necessary to optimize the performance of one of them to ensure the balance between them [19,20]. This requires us to consider resource allocation, interference coordination, etc., comprehensively. Under the premise of meeting the basic requirements of the system, it is particularly important to optimize and improve a certain indicator of communication or perception. With proper resource allocation and interference coordination strategies, we can ensure that the overall performance of the communication system is improved while meeting the requirements of specific application scenarios [21,22].

The ultimate goal of communication and perception integration is to realize the perfect integration of wireless perception and wireless communication in the same system, where the two functions can assist each other and achieve balance [23,24]. Promoting the development of 6G communication and sensing integrated systems is not only a simple integration of communication and sensing functions on the same hardware platform, but also a multi-level and comprehensive fusion process, involving multiple dimensions such as spectrum resources, beamforming, signal processing, protocol interfaces, and networking cooperation [25,26].

To realize the harmonious coexistence of communication and perception, we must consider the dual needs of the two and optimize the design of the system [27]. By integrating the sensing technology, the communication system can accurately adjust and configure the parameters of each node, thus greatly improving the transmission efficiency [28,29]. Therefore, ISAC technology will become the core technology of 6G communication, providing solid support for the construction of a new information infrastructure, which is characterized by intelligent connections and virtual–real integration [30,31].

With the continuous development of this technology, future communication will move towards a new era of more efficient and intelligent systems, offering greater convenience and possibilities in both personal and professional contexts [32,33].

In this paper, we describe the power allocation problem in ISAC systems as a constrained optimization problem and propose an innovative power allocation scheme to maximize the sum rate of users in the range. This scheme mainly considers user mobility and inter-user interference to optimize power allocation. In traditional ISAC communication, user mobility can significantly affect channel conditions, causing changes in path loss, Doppler shift, and propagation delay, thus affecting the communication rate and sensing accuracy. Moreover, in distributed systems, interference from multiple users presents a key challenge, reducing communication throughput and sensing accuracy. Unlike traditional methods, the proposed scheme can dynamically allocate resources according to user movements and explicitly incorporate interference into its optimization framework, which significantly reduces the influence of interference and enhances the robustness of the performance in dense user scenarios. This design ensures that the system maintains high-performance communication in multi-user dynamic scenarios. The main contributions are summarized as follows:

- Joint Optimization of Communication and Sensing: This paper proposes a novel approach to optimize both communication performance and sensing accuracy simultaneously. Traditional methods usually adopt a two-step optimization strategy, that is, optimization of the communication performance independently, and then optimization of the sensing performance or vice versa, which makes it difficult to take into account the interaction between the two in the optimization process. It is easy to produce suboptimal overall performance. Differing from this approach, the proposed method dynamically coordinates the power allocation strategy by considering both communication and sensing performance indicators in a single optimization framework, so as to realize the true joint optimization of communication and sensing performance and ensure coordination and optimality of the overall system.
- Advanced Interference and Fading Adaptation: The work introduces an interference-aware power control strategy and a fading-aware resource allocation mechanism that effectively reduces interference and adapts to fading channels, ensuring robust communication and efficient power allocation. The proposed scheme outperforms existing methods in terms of communication rates and interference management, particularly under severe multipath fading conditions. In addition, the joint optimization framework helps to avoid wastage of power resources, ensures that limited power resources are optimally allocated between communication and sensing tasks, and improves the overall efficiency of power utilization.

The remainder of this paper is structured as follows. Section 2 presents the system model and the formulation of the problem. Section 3 details the proposed optimization scheme for sensing-aided communication. Section 4 provides simulation results and performance analysis, comparing our proposed scheme with three comparison schemes. Section 5 concludes the paper and outlines future research directions.

## 2. System Model and Problem Formulation

**Figure 1:**
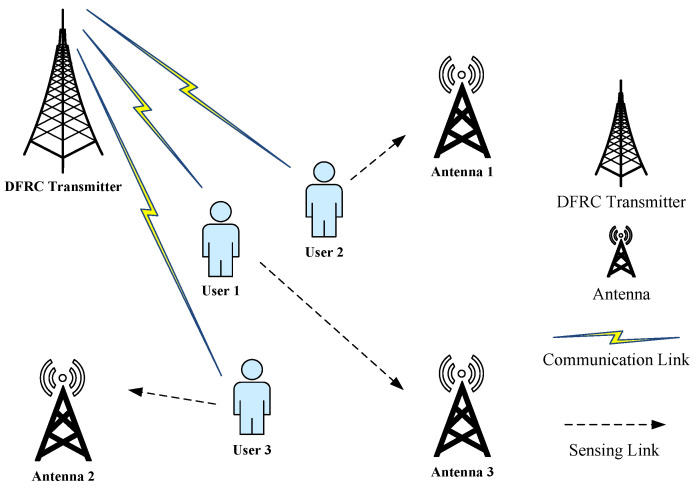
Schematic of the system model.

In this paper, we describe a distributed ISAC radar scenario consisting of *M* transceiver antennas and *N* users moving in random directions with velocity $v_{n}$, where $n=\left\{1,2,\ldots ,N\right\}$. In this model, each antenna serves all users at the same time and satisfies both communication requirements and sensing requirements, as shown in Figure 1.

### 2.1. Mobility Model

The transmitted signal of each antenna at moment *t* is $s_{m}\left(t\right)$, which $m=\left\{1,2,\ldots ,M\right\}$, received signal is $s_{m}\left(t-\tau \right)e^{j\varphi }$, the antenna relative to the user’s distance at *t* moment can be denoted by $d_{mn}\left(t\right)$, calculate as follows

(1) $$d_{mn}\left(t\right)=\frac{\tau c}{2}$$

where $n=\left\{1,2,\ldots ,N\right\}$, τ is the propagation delay of the signal from transmission to return, and *c* is the speed of light. The speed of the antenna relative to the user at moment *t* is $v_{mn}\left(t\right)$, which is calculated as follows

(2) $$v_{mn}\left(t\right)=\frac{\varphi \lambda }{4\pi T_{c}}$$

where φ is the phase difference of the transmitted signal with respect to the received signal, λ is the wavelength of the transmitted signal, and $T_{c}$ is the transmission time interval of adjacent transmitted signals.

The distance and speed that the antenna relative to users at moment *t* are $d_{mn}\left(t\right)$ and $v_{mn}\left(t\right)$, relative to the base station equipment at moment (t+Δt) and the distance is d(t+Δt). The user’s motion direction θ(t) with respect to the antenna at moment *t* can be calculated by the following equation.

(3) $$\cos\left[\pi -\theta \left(t\right)\right]=\frac{d{\left(t\right)}^{2}+{\left[v(t)\cdot \Delta t\right]}^{2}-d{\left(t+\Delta t\right)}^{2}}{2v(t)\cdot \Delta t\cdot d(t)}.$$

### 2.2. Signal Power Transmission Model

Suppose that at moment *t* the horizontal angle between the user and the antenna is $\theta_{mn}\left(t\right)\in \left[0,\pi \right]$. Considering that the user moves back in a random direction when the user moves away from the antenna, the antenna will allocate more power to the user. The direction angle is $\theta {\left(t\right)}_{mn}\in \left[0,\frac{\pi }{2}\right]$; As the user moves closer to the antenna, the antenna will allocate less power to the user with an Angle of $\theta {\left(t\right)}_{mn}\in \left[\frac{\pi }{2},\pi \right]$. It is calculated as follows

(4) $$p_{mr}\left(t\right)=p_{mt}\left(t\right)\cdot \frac{1}{4\pi^{2}{\left[d{\left(t\right)}_{mn}\right]}^{2}}\cdot \exp\left[\frac{c-v_{n}\left(t\right)\cdot cos\left[\theta_{mn}\left(t\right)\right]}{c}-1\right]$$

where $p_{mt}\left(t\right)$ is the transmission power at moment *t* for antenna *m*, $p_{mr}\left(t\right)$ is the received power at moment *t* for antenna *m*.

### 2.3. Signal Multipath Fading Model

In the actual situation, two IoT devices operating in the same frequency band will interfere with each other. Supposing that there will also be interference between any two users in this situation, user *x*, and user *y* are two different users in the environment, then the multipath fading influence of user *y* on user *x* is $I_{xt}\left(t\right)$

(5) $$I_{xy}\left(t\right)=k\left(t\right)\cdot \left[\exp\left(-\frac{v_{y}\left(t\right)\cos\left[\theta_{xy}\left(t\right)\right]}{c}\right)+\exp\left(-\frac{v_{y}\left(t\right)\cos\left[\theta_{xy}\left(t\right)\right]}{c}\right)\right]$$

where k(t) is the scaling factor. Its value is calculated as follows

(6) $$k\left(t\right)=\frac{p_{xy}\left(t\right)}{4\pi^{2}{\left[d_{xy}\left(t\right)\right]}^{2}c}.$$

$d_{xy}$ is calculated by the following equation

(7) $$d_{xy}=\sqrt{{\left[d_{x}\left(t\right)\right]}^{2}+{\left[d_{y}\left(t\right)\right]}^{2}-2d_{x}\left(t\right)\cdot d_{y}\left(t\right)\cos\left[\alpha_{xy}\left(t\right)\right]}$$

where $\alpha_{xy}\left(t\right)$ is the angle between user *x* and user *y* at *t* moment with respect to the pitch viewing plane of the base station.

After calculating the multipath fading influence between any two users, the multipath fading influence suffered by any user within the system model is

(8) $$I_{x}\left(t\right)=\sum_{y=1,x\neq y}^{N}I_{xy}\left(t\right)$$

## 3. Problem Analysis and Algorithm Analysis

### 3.1. Establishment of Constraint Model

This subsection takes the two starting points of user mobility and inter-user interference. The power transmission model and the signal multipath fading model are reconstructed. The ultimate goal was to find an optimal antenna power allocation scheme to maximize the sum rate of all users under the premise of satisfying the user-sensing performance constraints. The problem model was as follows

(9) $$\begin{array}{cc} \max\; & \sum_{n=1}^{N}R_{n}\\ s.t.\;\; & \sum_{n=1}^{N}p_{t_{n}}\left(t\right)=P_{T}\\ \; & P_{d_{n}}\left(t\right)\geq P_{thr_{n}}\left(t\right),\forall n\in \left\{1,2,\ldots ,N\right\}\\ \; & p_{min}\leq p_{t_{n}}\left(t\right)\leq p_{max},\forall n\in \left\{1,2,\ldots ,N\right\} \end{array}$$

The constraint conditions of the problem are power constraint and detection probability constraint, and the overall problem is formulated as obtaining the maximum communication rate under a fixed power. The minimum power limit $p_{min}$ in the constraints is mainly to ensure that even in the case of low communication demand, each user can obtain sufficient signal strength to maintain the basic communication link stability and avoid complete signal loss. The maximum power limit $p_{max}$ is mainly to avoid excessive power concentration on one user, leading to serious degradation of communication quality of other users, and to prevent adjacent channel interference caused by high power transmission of a single user.

The $P_{thr_{n}}\left(t\right)$ represents the minimum sensing power required by the *n*-th user at *t* moment to satisfy the detection probability threshold. The constraint of detection probability is set to meet the basic sensing performance requirements to assist the power allocation to users. In typical radar communication systems, higher detection probability means higher reliability of target detection and state perception, but it also means higher power consumption.

In Equation (9), $R_{n}$ is the communication rates of User *n*, which is calculated as follows

(10) $$R_{n}=\int_{t}^{t+\Delta t}\left(\log_{2}\left[1+\frac{p_{r_{n}}\left(t\right)}{I_{n}\left(t\right)}\right]\right)dt$$

where $p_{r_{n}}\left(t\right)$ is the received signal power of user *s* at *t* moment, In is the multipath fading influence of user *n* at *t* moment, calculated as by Equation (8). $P_{thr_{n}}\left(t\right)$ is the threshold of detection probability in Equation (9), and $P_{d_{n}}$ is the detection probability of user *n*. The derivation of the $P_{d_{n}}$ is carried out below. We first consider performing a Neyman–Pearson binary decision on the echo *y* within a single coherent time as follows

(11) $$\left\{\begin{array}{c} H_{0}:y∼CN\left(0,\sigma_{n}^{2}\right),\\ H_{1}:y∼CN\left(s_{n},\sigma_{n}^{2}\right), \end{array}\right.$$

where $H_{0}$ means that *y* is the zero-mean complex Gaussian noise in the presence of no detected target, and $H_{1}$ means that *y* is the complex Gaussian variable of the mean of the echo signal in the presence of target and $s_{n}$ is the mean of the random echo.

The echo power is $p_{r_{n}}={\left|s_{n}\right|}^{2}$. The incoherent energy detector is adopted, so the energy statistics is

(12) $$T={\left|y\right|}^{2}.$$

Under the two assumptions of H, *T*, respectively, follows the central and non-central chi-square distributions with 2 degrees of freedom. Its conditional probability density function $f_{T|H}\left(t\right)$ is

(13) $$f_{T|H_{0}}\left(t\right)=\frac{1}{\sigma_{n}^{2}}e^{-t/\sigma_{n}^{2}},\;f_{T|H_{1}}\left(t\right)=\frac{1}{\sigma_{n}^{2}+S_{n}}e^{-t/(\sigma_{n}^{2}+S_{n})}.$$

We set a fixed false alarm probability $P_{fa}$, with a threshold λ of

(14) $$\lambda =-\sigma_{n}^{2}\ln P_{fa}.$$

Thus, the detection probability is

(15) $$P_{d_{n}}=\mathrm{Pr}\left\{T>\lambda |H_{1}\right\}=\exp\left(\frac{\ln P_{fa}}{1+{\mathrm{SINR}}_{n}}\right)=P_{fa}^{1/(1+SNR_{n})}.$$

Then taking $P_{fa}=e^{-1}$, we can obtain

(16) $$P_{d_{n}}=\exp\left(-\frac{1}{1+{\mathrm{SINR}}_{n}}\right).$$

In Equation (16), the $\sigma_{n}^{2}$ is the noise power, and the ${\mathrm{SINR}}_{n}$ is the ${\mathrm{SINR}}_{n}$ of the echo signal, which is calculated as follows

(17) $${\mathrm{SINR}}_{n}=\frac{p_{r_{n}}}{I_{n}}=\frac{g_{nn}\;p_{t_{n}}}{\sigma_{n}^{2}+\sum_{j\neq n}g_{jn}\;p_{t_{j}}},$$

where $g_{nn}$ represents the channel gain from the *n*-th transmitter to the *n*-th receiver, and $u_{n}$ is the auxiliary receiving filter variable for the *n*-th user. So we can derive $p_{r_{n}}=g_{nn}p_{t_{n}}$ represents the effective power received by the *n*-th user and $I_{n}=\sigma_{n}^{2}+\sum_{j\neq n}g_{jn}p_{t_{j}}$ represents the total interference and noise of each channel.

Supposing that the $\gamma_{n}$ is the minimum SINR threshold given by the inverse function of the detection probability to ensure acceptable sensing performance, it has the following relationship

(18) $${\mathrm{SINR}}_{n}\geq \gamma_{n}.$$

The optimization problem constructed above has multiple constraints, and we choose to use the Karush–Kuhn–Tucker (KKT) conditions and construct the Lagrange equation to solve the model. The Lagrange function is constructed as follows

(19) $$\begin{array}{cc} Z\left[p_{r_{n}}\left(t\right),\mu ,\left\{\alpha_{n}\right\},\left\{\beta_{n}\right\},\left\{\lambda_{n}\right\}\right]= & -R_{n}+\mu \left(P_{T}-\sum_{n=1}^{N}p_{t_{n}}\left(t\right)\right)+\alpha_{n}\left[P_{thr_{n}\left(t\right)}-P_{d_{n}\left(t\right)}\right]\\ \; & +\beta_{n}\left[p_{min}-p_{t_{n}}\left(t\right)\right]+\lambda_{n}\left[p_{t_{n}}\left(t\right)-p_{max}\right]. \end{array}$$

The system of KKT equations consists of Equations (9) and (10) and the following formulas.

(20) $$\frac{\partial Z\left[p_{r_{n}}\left(t\right),\mu ,\left\{\alpha_{n}\right\},\left\{\beta_{n}\right\},\left\{\lambda_{n}\right\}\right]}{\partial p_{r_{n}}\left(t\right)}=0,n=\left\{1,2,\ldots ,N\right\},$$

(21) $$\mu ,\left\{\alpha_{n}\right\},\left\{\beta_{n}\right\},\left\{\lambda_{n}\right\}\geq 0,$$

(22) $$\alpha_{n}\left[P_{thr_{n}}\left(t\right)-P_{d_{n}}\left(t\right)\right]=0,$$

(23) $$\beta_{n}\left[p_{min}-p_{t_{n}}\left(t\right)\right]=0,$$

(24) $$\lambda_{n}\left[p_{t_{n}}\left(t\right)-p_{max}\right]=0.$$

### 3.2. Convergence and Complexity Analysis

Our analysis reveals that this optimization problem is a non-convex optimization problem. Because the constraints of this optimization problem are all linear constraints, the optimized target communication rate function has a nonlinear fractional structure, which is a logarithmic function. The independent variable of this objective function is SINR, and SINR is a fractional function of the power of each user. This renders the entire optimization objective no longer convex. Therefore, this optimization problem cannot be directly solved by applying the standard convex optimization theory.

For this non-convex optimization problem, we propose an engineering-feasible solution method which combines Weighted Minimum Mean Square Error with Successive Convex Approximation (WMMSE-SCA) (Algorithm 1). First, we rewrite the original objective function as

(25) $$\max_{p}\sum_{n=1}^{N}\log_{2}\left(1+\frac{p_{r_{n}}}{I_{n}}\right),$$

where $p={\left[p_{t_{1}},\ldots ,p_{t_{N}}\right]}^{⊤}$ represents the transmission power vector.

Construct Equation (25) equivalent form of least mean square error (MSE). By introducing the receiving filter $u_{n}\in C$ and the weight variable $w_{n}$, it can be proved that Equation (25) is equivalent to the following Equation [34]

(26) $$\min_{p,u,w}\sum_{n=1}^{N}w_{n}\varepsilon_{n}-\log_{2}w_{n},$$

where $\varepsilon_{n}$ is the weighted mean square error of the *n*-th user, defined as

(27) $$\varepsilon_{n}=|1-u_{n}\sqrt{p_{r_{n}}}{|}^{2}+{\left|u_{n}\right|}^{2}I_{n}.$$

Next, the SCA method is used to solve Equation (26). An iterative update approach is adopted. In each round, some variables are fixed to optimize the remaining variables, and u and w are iterated. When p is fixed, for each user, the MSE subproblem has the following closed-form solutions

(28) $$u_{n}^{*}=\frac{\sqrt{p_{r_{n}}}}{I_{n}},\;w_{n}^{*}=\frac{1}{\varepsilon_{n}^{*}},$$

where $\varepsilon_{n}^{*}$ is the MSE under $u_{n}^{*}$

The Taylor expansion linearization processing is then performed on the relevant terms of $\sqrt{p_{r_{n}}}=\sqrt{g_{nn}p_{t_{n}}}$ in the objective function to obtain the convex approximation terms

(29) $${\tilde{\varepsilon }}_{n}=1-2u_{n}\sqrt{g_{nn}}\left(p_{t_{n}}^{\left(k\right)}+\frac{p_{t_{n}}-p_{t_{n}}^{\left(k\right)}}{2\sqrt{p_{t_{n}}^{\left(k\right)}}}\right)+{\left|u_{n}\right|}^{2}\left(g_{nn}p_{t_{n}}+I_{n}\right).$$

Substituting the linearized WMMSE back into the objective function, the original problem degenerates into convex quadratic programming (QP). The new optimization problem is as follows.

(30) $$\begin{array}{cc} \min_{\left\{p_{t_{n}}\right\}}\; & \sum_{n=1}^{N}w_{n}{\tilde{\varepsilon }}_{n}-\sum_{n=1}^{N}\log_{2}w_{n}\\ s.t.\;\; & \left(1+\gamma_{n}\right)g_{nn}p_{t_{n}}-\gamma_{n}\sum_{j\neq n}g_{jn}p_{t_{j}}\geq \gamma_{n}\sigma_{n}^{2}.\\ \; & \sum_{n=1}^{N}p_{t_{n}}=P_{T},\;p_{\min}\leq p_{t_{n}}\leq p_{\max} \end{array}$$

Finally, when ${∥\nabla_{\;p}Z\left(p^{\left(k+1\right)}\right)∥}_{\infty }<\varepsilon_{g}$, the algorithm can be considered to converge and the iteration terminates, where $\varepsilon_{g}$ is the preset gradient threshold. This means that when the maximum gradient component of the current solution is less than the tolerance error $\varepsilon_{g}$, the power vector $p^{\left(k+1\right)}$ meets the accuracy requirements of the KKT gradient condition, which can be regarded as reaching the near global optimal solution and the iteration stops. The pseudo-code of the algorithm solution process is as follows.

**Figure 2:**
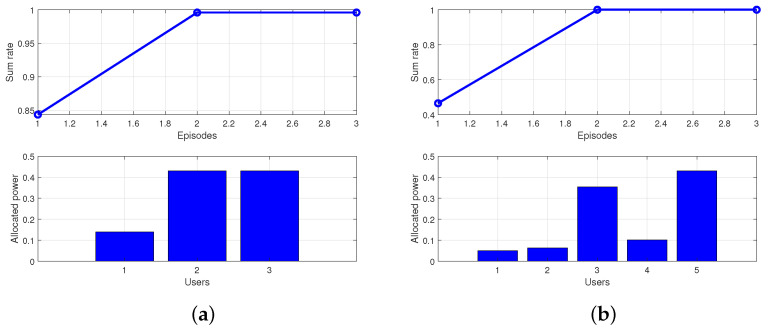
Performance of the WMMSE-SCA algorithm. (**a**) Three Users. (**b**) Five Users.

In numerical simulation, this solution method can obtain the near-global optimal solution after two rounds of iteration in the vast majority of cases. In a few cases, it requires three rounds of iteration to obtain the near-global optimal solution. The numerical simulation results are shown in Figure 2. We use the interior point method to solve this optimization problem, the time complexity of the problem is $O(N^{3})$. Examples of three users versus five users were selected, where the power of users is the normalized power and the sum rate is the dimensionless constant.
**Algorithm 1** WMMSE-SCA Algorithm  1:Initialization: Initializing transmit power vector $p={\left[p_{t_{1}},\ldots ,p_{t_{N}}\right]}^{⊤}$ to satisfy total power and SINR constraints;  2:Set auxiliary variables $u={\left[u_{t_{1}},\ldots ,u_{t_{N}}\right]}^{⊤}$ and $w={\left[w_{t_{1}},\ldots ,w_{t_{N}}\right]}^{⊤}$ arbitrarily;**Repeat**  3:Update $u_{n}\leftarrow \sqrt{p_{r_{n}}}/I_{n}$;  4:Update $w_{n}\leftarrow 1/\varepsilon_{n},\;$ where $\;\varepsilon_{n}=|1-u_{n}\sqrt{p_{r_{n}}}{|}^{2}+{\left|u_{n}\right|}^{2}I_{n}$;  5:Reformulate the SINR constraint using the new CSI where $\frac{\left(g_{nn}-\varepsilon_{nn}\right)p_{t_{n}}}{\sigma_{n}^{2}+\sum_{j\neq n}\left(g_{jn}+\varepsilon_{jn}\right)p_{t_{j}}}\geq {\tilde{\gamma }}_{n}$;  6:Solve the resulting convex QP problem over p with fixed u, w;**Until convergence**

In order to evaluate the engineering value of this algorithm, we make a simple estimate of the solution time of the algorithm. Assuming there are a total of 32 users in the region, the algorithm needs to be iterated three times to converge, and the requirement for calculation accuracy is $10^{-3}$. Then, the amount of floating-point calculations is approximately $5\times 10^{4}$ times. If using ARM Cortex-M series chips, such as the STM32F407 series single-chip microcontrollers, their single-core computing capacity is approximately 90 MFLOPS, and the required computing time is approximately 4.5 ms. This means that the scheme can fully satisfy the requirements of 6G communications or Internet of Vehicles technology.

### 3.3. Performance and Robustness Analysis

In this section, the theoretical rate upper bound of the proposed scheme is discussed, and the potential presence of errors in the motion state of the sensing user in the actual scenario is considered. We also discuss the robustness of the scheme in the presence of sensing data delay or serious noise, and the strategy of the scheme in the case of imperfect channel state information (CSI) or outdated due to feedback delay is analyzed.

We first derive the sum rate upper bound of the proposed scheme. We assume that the upper bound is only given by completely eliminating the inter-user interference and relaxing the detection probability constraint, subject to the total power constraint. In the initiation case of $g_{jn}=0,\;j\neq n$, all channels in the system degenerate into AWGN channels. The capacity of the *n*-th channel can be formulated as

(31) $$C_{n}\left(p_{n}\right)=\log_{2}\;\left(1+\frac{g_{nn}p_{n}}{\sigma_{n}^{2}}\right).$$

According to the water level allocation principle [35], the maximum sum rate under the total power constraint $\sum_{n}p_{n}=P_{T}$ is

(32) $$\bar{C}=\max_{\begin{array}{c} \sum_{n}p_{n}=P_{T}\\ p_{n}\geq 0 \end{array}}\sum_{n=1}^{N}C_{n}\left(p_{n}\right)=\sum_{n=1}^{N}\log_{2}\;{\left(\frac{g_{nn}}{\sigma_{n}^{2}}\mu \right)}^{+},$$

where the water level μ is given by $\sum_{n}{\left(\mu -\sigma_{n}^{2}/g_{nn}\right)}^{+}=P_{T}$ uniquely determined. For any scheme with mutual interference or additional constraints, the achievable rate must not exceed $\bar{C}$, which is the upper bound of the scheme.

**Figure 3:**
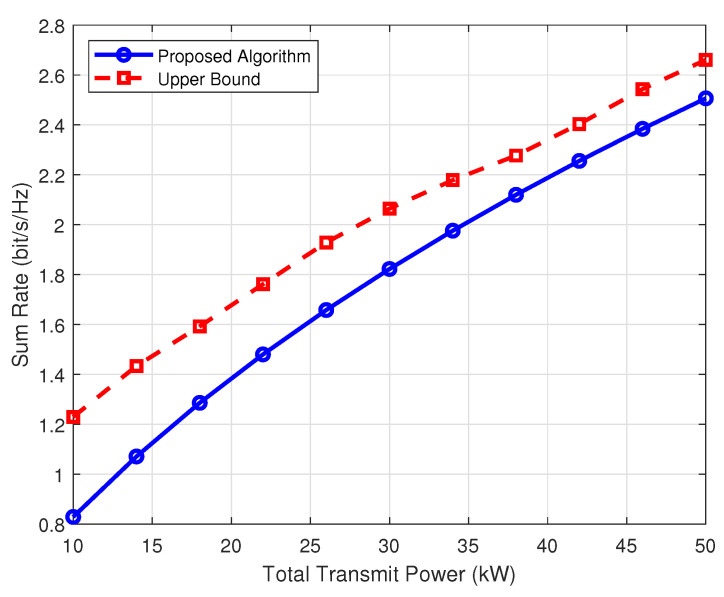
Comparison of actual sum rate and upper bound rate of the proposed scheme.

A comparison between the performance of the proposed scheme and the upper bound of the rate is shown in Figure 3. It can be seen that in the case of low transmission power, the influence of inter-user interference and Gaussian white noise is great, and the actual sum rate of the scheme can reach 70% of the upper bound of the rate. With the continuous increase of the transmission power, the sum rate is closer to the upper bound of the rate. The performance of the proposed scheme can achieve 95% of the upper bound.

The following is the robustness analysis of the scheme in the case of noise or delay in the sensing data in the actual scene. Assume that the actual motion state of the user is

(33) $$v_{n}\left(t\right)={\hat{v}}_{n}\left(t\right)+\Delta v_{n}\left(t\right),$$

(34) $$\theta_{n}\left(t\right)={\hat{\theta }}_{n}\left(t\right)+\Delta \theta_{n}\left(t\right),$$

where ${\hat{v}}_{n}\left(t\right)$, ${\hat{\theta }}_{n}\left(t\right)$ is the sensing estimator and $\Delta v_{n}\left(t\right)$, $\Delta \theta_{n}\left(t\right)$ is the estimation error. When there is an error in the state information of the sensing user, it may cause our estimation of the detection probability function to be too high, which may lead to the SINR constraint $\frac{p_{r_{n}}}{I_{n}}\geq \gamma_{n}$ being mistakenly relaxed, resulting in the overall performance degradation of the system. At this point, after determining that there is sensing uncertainty, the detection probability constraint is modified as

(35) $$\min_{∥\Delta v_{n}∥\leq \varepsilon_{v},\;∥\Delta \theta_{n}∥\leq \varepsilon_{\theta }}\;P_{d_{n}}\left(v_{n},\theta_{n},{\mathrm{SINR}}_{n}\right)\;\geq \;P_{{\mathrm{thr}}_{n}},$$

where $P_{d_{n}}$ is a monotonically decreasing function with $v_{n}$ and $\theta_{n}$. Then $v_{n}$ and $\theta_{n}$ in the case of maximum sensing error are $v_{n}={\hat{v}}_{n}+\varepsilon_{v}$ and $\theta_{n}={\hat{\theta }}_{n}\pm \varepsilon_{\theta }$, in which case we replace γ with the more conservative $\tilde{\gamma }$, and the new robust sensing constraint is

(36) $$\frac{p_{r_{n}}}{I_{n}}\geq {\tilde{\gamma }}_{n},\;{\tilde{\gamma }}_{n}>\gamma_{n}.$$

The problem of user state information error can be solved by replacing the sensing constraint in Equation (9) with Equation (35).

In the case of imperfect channel state Information (CSI) or errors or delays in CSI, consider that there is an estimation error $\Delta g_{jn}$ for the gain of each user to all links $g_{jn}$, denoted by

(37) $$g_{jn}={\hat{g}}_{jn}+\Delta g_{jn},\;\Delta g_{jn}\in \left[-\varepsilon_{jn},+\varepsilon_{jn}\right]$$

where the estimation error of $g_{jn}$ will affect $I_{n}$ and $p_{r_{n}}$, and then the SINR under poor CSI is lower bounded by

(38) $${\mathrm{SINR}}_{n}^{\mathrm{worst}}=\frac{\left(g_{nn}-\varepsilon_{nn}\right)\;p_{t_{n}}}{\sigma_{n}^{2}+\sum_{j\neq n}\left(g_{jn}+\varepsilon_{jn}\right)\;p_{t_{j}}}.$$

When the lower bound is used for objective function constraints, it can be expressed as

(39) $$\frac{\left(g_{nn}-\varepsilon_{nn}\right)p_{t_{n}}}{\sigma_{n}^{2}+\sum_{j\neq n}\left(g_{jn}+\varepsilon_{jn}\right)p_{t_{j}}}\geq {\tilde{\gamma }}_{n}.$$

By linearizing this constraint, we obtain

(40) $$\left(1+{\tilde{\gamma }}_{n}\right)\left(g_{nn}-\varepsilon_{nn}\right)p_{tn}-{\tilde{\gamma }}_{n}\sum_{j\neq n}\left(g_{jn}+\varepsilon_{jn}\right)p_{tj}\geq {\tilde{\gamma }}_{n}\sigma_{n}^{2},$$

where it turns into a convex approximation constraint, which can be solved by WMMSE-SCA algorithm.

## 4. Performance and Simulation Analysis

We conduct a series of simulation experiments in which the Rayleigh channel and the Rician channel are considered in the simulation to evaluate the performance of the proposed sensing-sided communication method for distributed radar communication systems. The proposed scheme which jointly considers mobility and multi-user interference (JMMUI) is compared with the following three schemes to prove the effectiveness and advantages of the proposed scheme: (1) only the mobility of users is considered (OMU); (2) only the multi-user interference is considered (OMUI); (3) neither the mobility of users nor the mutual interference between users is considered (NC). The data for each parameter is set according to the actual situation. The initialization of the main parameters is shown in Table 1.

### 4.1. Different Detection Probability Constraints

**Figure 4:**
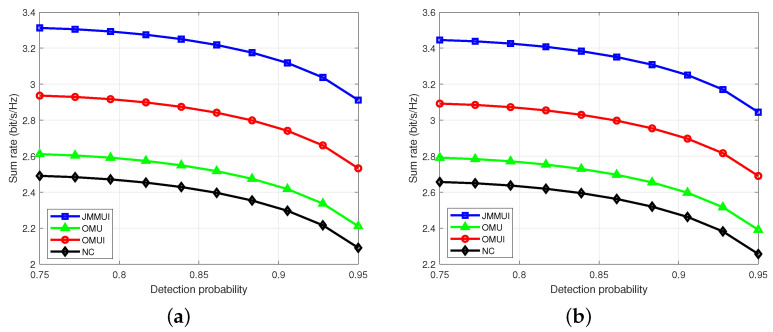
The sum rate under different detection probability constraints. (**a**) Rayleigh channel. (**b**) Rician channel.

Figure 4 depicts each scheme’s sum rate under different perceptual performance constraints. In general, the performance of the scheme in the Rayleigh channel is close to that in the Rician channel. However, due to the better conditions of the Rician channel, the sum rate in the Rician channel is higher than that in the Rayleigh channel as a whole, and the trend of decreasing with the increase in detection probability is smaller. The JMMUI scheme shows better performance than the other three comparison schemes under various detection probability constraints. This advantage is due to the comprehensive consideration of user mobility and inter-user interference in the JMMUI scheme. In contrast, the comparison OMU scheme and the OMUI scheme only pays attention to one aspect, and the comparison scheme NC completely ignores the influence of these two factors. Therefore, as shown in Figure 2, the JMMUI scheme offers more performance advantages. The sum rate decline trends and values of comparison OMU scheme and OMUI scheme under different sensing performance constraints are not much different, but both are better than the comparison scheme NC. In addition, with the increase of detection probability, the sum rate of all schemes shows a nonlinear decreasing trend. This phenomenon shows that as the constraints become more stringent, the optimal performance value that can be achieved decreases. This is because as the detection probability constraint becomes more stringent, more power is needed to be allocated for sensing, and the power allocated for communication decreases. In summary, the JMMUI scheme presents more advantages in considering the influence of user mobility and inter-user interference and shows better performance under different perceptual performance constraints. At the same time, the sum rate of all schemes shows a non-linear decreasing trend with increasing detection probability.

### 4.2. Different Total Power Constraints

**Figure 5:**
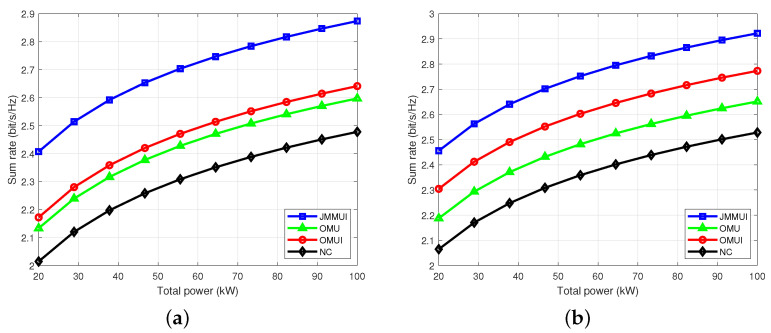
The sum rate under different total power constraints. (**a**) Rayleigh channel. (**b**) Rician channel.

Figure 5 depicts the sum rate that the user can achieve under different total power constraints. It can be seen that the performance of the proposed scheme under Rician channel is slightly better than that under Rayleigh channel. Through careful observation, it can be found that under various total power constraints, the JMMUI scheme shows superior performance over the other three comparison schemes. This excellent performance is due to the comprehensive consideration of user mobility and inter-user interference, whereas the OMU scheme and the OMUI scheme focus on only one aspect. In contrast, the contrast scheme NC completely ignores these two key factors. Therefore, the performance of the JMMUI scheme is significantly better than other schemes. The sum rate of the comparison OMU scheme and OMUI scheme under different total power constraints shows a similar upward trend, and the difference is not large in value, but both are better than the comparison scheme NC. In addition, as the total power increases, the sum rate of all schemes shows a non-linear upward trend. This trend indicates that as the total power increases, the constraints become more relaxed, providing more room for choice in the distribution, and thus achieving a higher performance optimal value. In summary, the JMMUI scheme has more advantages in considering the influence of user mobility and inter-user interference and shows excellent performance under different total power constraints, which also provides a valuable reference for future research and optimization.

### 4.3. Complex Urban Environment Simulation

**Figure 6:**
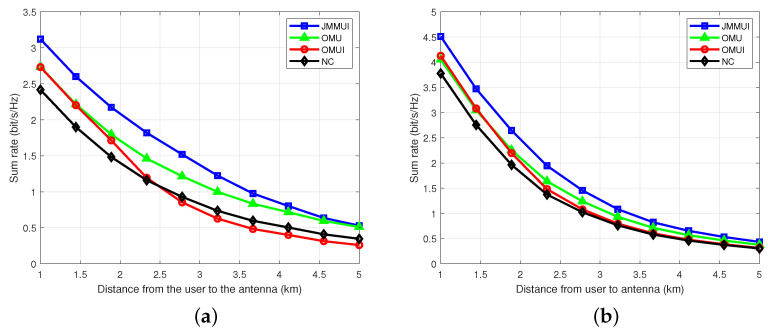
The relationship between the sum rate and the average distance between the user and the base station. (**a**) Rayleigh channel. (**b**) Rician channel.

To study the performance of the power allocation scheme proposed in this paper in a complex urban environment, we simulated the sum rate that can be achieved at the average distance of different multi-user distance antennas. The results are shown in Figure 6. In this scenario, the JMMUI scheme has significant performance advantages in the Rice channel. It is found that in the distance of 1∼5 km, the JMMUI scheme shows superior performance over the other three schemes. Due to the comprehensive consideration of user mobility and inter-user interference, contrast schemes OMU and OMUI only focus on one aspect, while contrast scheme NC completely ignores these two key factors. When distance increases, the sum rate of all schemes decreases, reflecting the fading effect of the signal during transmission. We can also find that when the distance is greater than 2.5 km, the users’ sum rate of OMUI scheme considering only inter-user interference is lower than that of scheme NC, which is reflected in the fact that when the user and the base station communicate over a long distance, the benefit of power consumption to consider the optimization of inter-user interference is very small. When the distance exceeds a critical point, it even has a negative return compared to the case of equal power allocation. For example, in this paper, scheme NC adopts the equal-sharing power strategy, which means that each user can obtain a relatively stable signal power allocation, even if the path loss increases, the power attenuation of all users is uniform, so the amplitude of their communication rates decline is relatively stable, while the OMUI scheme is still trying to reduce interference at a long distance due to the inter-user interference optimization strategy. However, if the signal power is not effectively enhanced, the overall signal quality decreases faster than scheme NC when the path loss is intensified. This problem is more significant in Rayleigh channels. In summary, the JMMUI scheme is more advantageous in considering the influence of user mobility and user-to-user interference and shows excellent performance at different average distances of multiple users from the antenna. At the same time, when the distance increases, the sum rate of each scheme shows a nonlinear decreasing trend.

**Figure 7:**
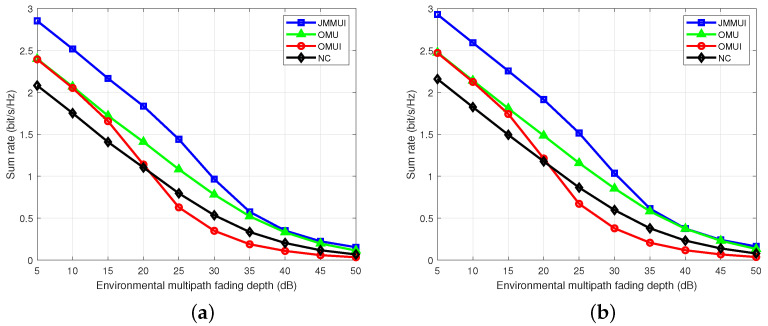
The relationship between the sum rate and the environmental depth of multipath fading. (**a**) Rayleigh channel. (**b**) Rician channel.

To study the performance of the power allocation scheme proposed in this paper in a complex urban environment, we simulated the sum rate that can be achieved under different multi-path fading depths in the environment. In this term, there is no significant difference between the Rayleigh channel and the Rice channel, with only a small sum rate advantage of the Rice channel fixed. The results are shown in Figure 7. It is found that, in a general environment with a fading depth of 5∼20 dB, the JMMUI scheme has significantly superior performance than the other three schemes. Even in a complex urban environment with a fading depth of 20∼50 dB that may occur, the JMMUI scheme has significantly improved performance compared with the other three schemes. Due to the comprehensive consideration of user mobility and inter-user interference, the OMU scheme and the OMUI scheme only focus on one aspect. In contrast, the contrast scheme NC completely ignores these two key factors. When the depth of environmental fading increases, the sum rate of all schemes decreases, reflecting the negative effect of the depth of environmental fading on the communication rates. We can also find that when the depth of the environmental fading is greater than about 20 dB, the sum rate of the OMUI scheme is lower than that of the scheme NC. This shows that the effect of the interference optimization is greatly restricted in a strong fading environment. At this time, the continuous optimization of inter-user interference will lead to unreasonable allocation of system resources, and thus reduce the sum rate. On the contrary, scheme NC allocates stable signal transmission power in a strong fading environment through resource allocation of equal power, thus it achieves a higher sum rate than the OMUI scheme. In summary, the JMMUI scheme has more advantages in considering the influence of user mobility and inter-user interference and shows excellent performance under different multi-path fading depths in different environments. At the same time, when the depth of multipath fading increases, the sum rate of each scheme shows a nonlinear decreasing trend.

### 4.4. Simulation Analysis Summary

In summary, compared with the three schemes, the proposed scheme shows excellent performance. Meanwhile, user mobility and inter-user interference are both important factors affecting the sum rate of the multi-user system built by dual-function radar. In the case of simple environments (weak interference), both user mobility and inter-user interference are considered and optimized to have similar effects on the sum rate. In the case of complex environments (strong interference), the optimization of user mobility becomes the main factor affecting the sum rate, while the optimization of inter-user interference has little impact on the sum rate. The scheme proposed in this paper, which comprehensively considers user mobility and inter-user interference, can achieve the sum rate in most cases.

## 5. Conclusions

In this paper, we proposed a perception-aided communication method for a distributed radar communication system. By taking into account the mobility of users and the interference between users, the communication performance of the system is improved. Excellent joint improvement of communication and sensing, and advanced interference and fading adaptation are obtained by optimizing the power allocation scheme. The rigorous experimental simulations demonstrate that the proposed method significantly improves the sum rate, especially when compared to schemes that only address user mobility or interference individually. The results underline the value of integrated sensing and communication for future applications, particularly in the context of 6G networks, which aim to merge sensing and communication in a unified system. The study not only advances the efficiency of distributed radar communication systems but also paves the way for future work involving machine learning and real-time applications to further improve system performance.

This work contributes to the growing field of ISAC by offering an adaptive resource allocation framework that ensures reliable and efficient communication in distributed radar communication systems. The insights gained from this research can serve as a foundation for further optimization techniques and the development of future 6G networks that seamlessly combine communication and sensing functions. In future work, we aim to extend the proposed framework by integrating machine learning-based interference prediction and adaptive beamforming techniques to further enhance system performance in more complex and dynamic environments. Additionally, real-time hardware implementation and network scalability will be explored to validate the feasibility and practicality of the proposed solution in large-scale, high-density networks.

## Figures and Tables

**Table 1:** The values of the main parameters.

Parameter	Value	Units
The coordinates of antenna 1	[4,3]	$\times 10^{3}m$
The coordinates of antenna 2	[−4.5,4.5]	$\times 10^{3}m$
The coordinates of antenna 3	[−5,−4.5]	$\times 10^{3}m$
The coordinates of antenna 4	[4,−5]	$\times 10^{3}m$
The coordinates of user 1	[4,5]	$\times 10^{3}m$
The coordinates of user 2	[−3,3]	$\times 10^{3}m$
The coordinates of user 3	[4.5,−4.5]	$\times 10^{3}m$
The speed of users	[5,4,6]	×km/h
Angle of motion direction relative to X-axis	$\left[\frac{\pi }{4},\frac{3\pi }{4},\frac{7\pi }{4}\right]$	rad

## Data Availability

The original contributions presented in this study are included in the article. Further inquiries can be directed to the corresponding author.
